# The Spectrum of Neurological Manifestations Associated with Gaucher Disease

**DOI:** 10.3390/diseases5010010

**Published:** 2017-03-02

**Authors:** Tamanna Roshan Lal, Ellen Sidransky

**Affiliations:** Medical Genetics Branch, National Human Genome Research Institute, National Institutes of Health, Building 35A Room 1E623, 35A Convent Drive, Bethesda, MD 20892-3708, USA; tamanna.roshanlal@nih.gov

**Keywords:** Gaucher disease, neuronopathic, parkinsonism, glucocerebrosidase gene (*GBA1*), glucocerebrosidase, myoclonic epilepsy

## Abstract

Gaucher disease, the most common lysosomal storage disorder, is due to a deficiency in the enzyme glucocerebrosidase. This leads to the accumulation of its normal substrate, glucocerebroside, in tissue macrophages, affecting the hematological, visceral, bone and neurologic systems. Gaucher disease is classified into three broad phenotypes based upon the presence or absence of neurological involvement: type 1 (non-neuronopathic), type 2 (acute neuronopathic), and type 3 (subacute neuronopathic). Phenotypically, there is a wide spectrum of visceral and neurological manifestations. Enzyme replacement is effective in managing the visceral disease; however, treating the neurological manifestations has proved to be more challenging. This review discusses the various neurological manifestations encountered in Gaucher disease, and provides a brief overview regarding the treatment and ongoing research challenges.

## 1. Introduction

Recent progress in human genetics has resulted in dramatic advances in our understanding of “simple” recessive disorders. This has provided unique insight into the complexities of both Mendelian and more complex disorders. Clinical research of one such monogenic disorder, Gaucher disease (GD), has shown that it manifests with broad phenotypic variation, ranging from neonatal lethality to asymptomatic octogenarians [1]. There are some forms that spare and others that affect the central nervous system (CNS) in different ways. Neurological involvement can occur very early on in the disease process, resulting in severe neurodegeneration and death in infancy or early childhood, or manifestations can present later in life. This vast heterogeneity has at times made the disorder difficult to discern, leading to delays in diagnosis and management. GD has been classified into three different subtypes based on the absence (type 1), or the presence and severity of neurological involvement (types 2 and 3).

Non-neuronopathic type 1 GD (OMIM #230800) is the most common form, accounting for approximately 94% of cases in the Western world. Clinical manifestations include hepatosplenomegaly, splenomegaly, anemia and thrombocytopenia. Clinical or radiographic evidence of bone disease (osteopenia, focal lytic or sclerotic lesions, and osteonecrosis) as well as pulmonary involvement can also be seen. It has been reported that type 1 GD is associated with an increased risk of certain malignancies, especially multiple myeloma [2,3]. The extent of symptoms is highly variable, and many affected individuals never receive or seek medical attention [4]. It is a panethnic disorder, but is especially prevalent among individuals of Ashkenazi Jewish descent. In recent years, the phenotype has expanded with the recognition of a subset of patients with GD who developed Parkinsonism, and in fact, there are now many patients who receive the diagnosis of Gaucher disease from a Parkinson’s clinic [5]. 

Type 2 GD (OMIM #230900), the acute neuronopathic form, is also the most severe form of GD. Traditionally, the clinical presentation is considered to be stereotypic; however, it can range from hydrops fetalis to the collodion baby phenotype to infants presenting after 6 months of life. Uniformly, there is rapid progression with severe neurodegeneration, leading to death in infancy or early childhood [1,6]. Type 3 GD (OMIM #2301000), the chronic form of this disorder, encompasses multiple different phenotypes. However, the hallmark clinical abnormality seen in type 3 GD consists of markedly slow horizontal saccades [7,8]. 

In certain situations, the boundaries between the different types of GD can become hazy. For example, although type 1 GD is considered non-neuronopathic, there have been instances where patients developed neurological symptoms as a result of spinal compression fractures. Peripheral neuropathies have also been described in patients considered to have type 1 GD. Also, a subset of patients develops an intermediate phenotype between types 2 and 3 of GD, with survival of up to 3–8 years [9]. It has been proposed that GD can be best viewed as having a continuum of phenotypes.

GD should be suspected when a patient presents with hepatosplenomegaly, thrombocytopenia, anemia, characteristic bone lesions, or signs of CNS involvement. The suspicion of type 1 GD should be higher when the patient is of Ashkenazi Jewish ancestry. In the past, Gaucher cells were identified in tissue biopsy specimens, principally bone marrow. However, this is no longer indicated, as the diagnosis can be made by measuring acid β-glucosidase activity in peripheral blood leukocytes. Enzymatic analysis can also be performed on fibroblasts cultured from skin biopsy specimens. Ultimately, confirmation of this disorder is done by molecular analysis of the glucocerebrosidase gene, *GBA1*. 

The glucocerebrosidase gene, *GBA1*, located on chromosome 1q21, encodes for the lysosomal enzyme glucocerebrosidase (E.C.3.2.1.45) [10,11]. This enzyme normally hydrolyzes glucocerebroside to glucose and ceramide. Mutations in *GBA1* cause a deficiency of glucocerebrosidase, leading to the accumulation of glucocerebroside in lysosomes of macrophages, ultimately resulting in multiorgan involvement. The ensuing clinical condition, first described by Phillippe Gaucher in 1882 [11], is the most prevalent recessively inherited lysosomal lipid storage disease. In a given family where the DNA genotype is known, or in laboratories that appropriately sequence all *GBA1* exons, diagnosis can be made by molecular diagnosis alone. 

Currently, there are two types of treatment approved for GD, enzyme replacement therapy (ERT) and substrate replacement therapy (SRT). ERT using recombinant glucocerebrosidase is used to reduce glucocerebroside levels and thus control visceral and hematological complications in patients with GD. ERT has proven to be important and effective in ameliorating the organomegaly, anemia and thrombocytopenia in patients with GD [12]. It also can prevent some of the bony manifestations. However, this treatment does not cross the blood–brain barrier, and is therefore ineffective in reversing neurological dysfunction in patients with GD disease types 2 and 3. SRT is an approach in which the glycolipid accumulation is counteracted by reducing the substrate level to better balance residual activity of the deficient enzyme [13].

The *GBA1* gene, organized in 11 exons, encodes for the 497 amino acid mature glucocerebrosidase (GCase) as well as a 19 amino acid signal peptide [14]. Mutations have been identified throughout the gene and include missense, stop and frame-shift mutations, as well as insertions and deletions. Several hundred mutations have been identified to date [15]. The presence of a highly homologous nearby pseudogene sequence complicates the molecular diagnosis for GD, as care must be taken to identify primers that exclusively amplify the functional gene. The pseudogene has been found to be the source of both point mutations and recombinant mutant alleles.

## 2. Epidemiology

GD is a common lysosomal storage disease throughout the world and the risk of developing GD increases in populations with consanguinous unions, inbreeding or geographic isolates. A recent review of the literature found that the worldwide incidence ranged from 0.39–5.80 per 100,000 with a prevalence of 1.33–1.75 per 100,000, although the data available is quite limited [16]. The carrier frequency for a glucocerebrosidase gene mutation is estimated at 0.0343 in the Ashkenazi Jewish population, and 0.006 in the general population [10,16]. The majority of patients have type 1 GD, the main type seen in the Ashkenazi Jewish population. Neuronopathic forms of GD are rarer, more severe variants of this disorder, with an estimated incidence of <1 in 100 00 live births [7]. Type 3 GD is mainly seen in Northern Europe, Egypt and East Asia, with a specific geographic isolate in the Norbottnian region of Sweden. In the Czech Republic, Netherlands and Portugal the standardized prevalence figures for types 2 and 3 GD combined are 0.34, 0.26 and 0.55 per 100,000 respectively. In Africa, Asia and the Middle East the incidence of neuronopathic GD might be higher than type 1, although accurate data is not available. 

## 3. Genotype–Phenotype Correlation in Gaucher Disease

Understanding genotype–phenotype correlation in GD has proven to be challenging, as individuals sharing the same genotype, even siblings or twins, can differ in their disease manifestations, clinical course and response to therapy [6,17]. For instance, phenotypes associated with genotype N370S/N370S range from children with significant organomegaly, growth delay or bone disease, to asymptomatic adults. However, patients carrying a N370S allele generally do not have neuronopathic GD, and N370S (also referred to as p.N409S) compound heterozygotes may have more severe disease manifestations than those with two N370S alleles. Another example where vast phenotypic heterogeneity is observed is with mutation L444P (p.L483P). Among 35 confirmed L444P homozygotes, residual enzymatic activity ranged from 1%–30%, unrelated to symptom severity. Furthermore, the patient phenotypes ranged from death in early childhood, to autism, to successful college students, implicating the role of genetic modifiers [2]. Even among Norrbottnian patients who share genotype L444P/L444P, significant clinical variation has been reported [18]. 

Homozygosity for recombinant or null alleles is associated with prenatal or perinatal lethality. Generally, genotype L444P/L444P is not seen in type 2 GD; however, the babies can appear to have two L444P alleles when one is a part of a recombinant allele arising from the pseudogene sequence [19].

A unique variant of type 3 GD is associated with homozygosity for mutation D409H (p.D448H). These patients can develop aortic fibrosis or calcifications as well as dysmorphic features and hydrocephalus, in addition to eye movement abnormalities [20,21]. The phenotype has been reported in patients from Jordan, Israel, Japan, Turkey and the USA. However, subjects homozygous for both D409H+H255Q (p.D448H+p.H294Q] in cis have type 2 GD [22,23,24].

### 3.1. Patients with Type 1 Gaucher Disease and Parkinsonism

Mutations in *GBA1* are important and common risk factors for Parkinson’s disease and related disorders [25,26]. This association was first established based on longitudinal clinical studies, whereby some patients with GD also developed Parkinsonism. Patients can exhibit an asymmetric onset of rigidity, resting tremor, and bradykinesia that is responsive to levodopa, while others have atypical, levodopa-resistant disease. It was later recognized that Parkinson’s disease is more frequent in first-degree relatives of patients with GD [26]. Studies in specific cohorts of patients with Parkinson’s disease and associated Lewy body disorders have indicated that these patients have an increased frequency of *GBA1* mutations compared to control groups [27,28]. Atypical features and non-motor manifestations have also been described in patients with *GBA1* mutations, such as supranuclear oculomotor signs, cognitive dysfunction, dementia, sleep disturbances, hallucinations and apraxia. In general, *GBA1-*associated Parkinsonism often has an earlier age of onset (mean approximately 4–5 years earlier) and more prominent cognitive impairment, although some patients do well over prolonged periods of time [25,29,30,31]. The basis for this association is unknown although different theories have been proposed [29,31]. It must be emphasized the vast majority of patients with GD and *GBA1* mutation carriers will never develop Parkinson’s disease. However, this association will likely prove useful in identifying other alleles that may place a person at increased or decreased risk of developing Parkinsonism. It also draws attention to the role of the lysosome in neurodegenerative disease. 

### 3.2. Type 2 (Acute) Neuronopathic Gaucher Disease

Type 2 GD is a progressive neurodegenerative disorder, usually resulting in death by age 1–3 years [1]. All patients with type 2 GD experience a rapid neurological decline, but manifestations vary widely. These patients present in early infancy with evidence of brainstem dysfunction consisting of supranuclear gaze palsy, irritability, hypertonia and/or hypokinesia [7]. This is followed by progressive and rapid deterioration associated with a convergent strabismus (bilateral sixth nerve palsy), dysphagia, stridor with breath holding episodes, pyramidal signs (cortical thumbs, retroflexion of the neck), failure to thrive and cachexia [9,32]. The brainstem deterioration seen in these children is fairly rapid and eventually leads to laryngeal obstruction/spasms, apnea, and dysphagia, often provoking aspiration. Other symptoms seen in this patient population include microcephaly, arthrogryposis, myoclonic jerks, seizures, cognitive impairment, rigidity, opisthotonos and profound developmental delay. A particularly lethal form of type 2 GD can be diagnosed prenatally with the identification of features of hydrops fetalis, a fetal condition characterized by edema and the accumulation of fluid in at least two different compartments. Another variant of type 2 GD is the “collodion baby” phenotype, where infants appear to be covered by a cellophane membrane at birth, and have a virtual absence of residual glucocerebrosidase activity. Clinical presentation of this variant include death at parturition, hydrops fetalis or death within the first few days of life [7]. Congenital ichthyosis may also be an associated feature of this phenotype. Often, a careful history reveals a pattern of recurrent fetal loss in the affected families.

### 3.3. Type 3 (Subacute, Chronic) Neuronopathic Gaucher Disease

Type 3 GD is a form presenting between infancy and adolescence and even rarely in adulthood [32], which is a continuum of different clinical presentations. Some patients with type 3 GD exhibit slowed horizontal saccadic eye movements as their sole neurological manifestation. Other patients have slowly progressive neurological disease with generalized or myoclonic seizures and mild to moderate organomegaly. Others have extensive organ involvement and bone disease with the early development of horizontal supranuclear palsy, but rarely develop progressive neurological disease. A less common form, characterized by cardiac and/or aortic calcifications or fibrosis, impaired saccadic eye movements, and, at times, hydrocephalus and skeletal anomalies, is associated with mutation D409H [1,9,33]. Some patients with type 3 GD have been found to have associated developmental delays, language difficulties, dementia and learning disabilities [9]. Cognitive deficits in patients with chronic neuronopathic GD typically affect general nonverbal skills, with below-average intellectual skills, and weaknesses observed in the areas of processing speed, visual-spatial relationships, and perceptual organization skills [34]. Verbal skills tend to be relatively spared. Some patients have remarkably high verbal IQ scores.

Although the neuronopathic forms are the rarest variants of GD, there is an endemic form of chronic neuronopathic GD among the Swedish population, specifically those living in northern Sweden in the county of Norrbotten. This form of GD affects approximately 40% of all known cases in Sweden. The Norrbottnian form is a well-characterized subtype of type 3 GD, with the first clinical symptoms occurring at the median age of 1 year. Symptoms seen with this form of the disorder include hematological and visceral symptoms, as well as a horizontal supranuclear gaze palsy with skeletal involvement, often including a gibbus deformity. Other manifestations include a convergent squint (due to abducens nerve palsy), retinal infiltrates, ataxia, mild spasticity in the legs, epilepsy (myoclonic or complex partial seizures), and a slow cognitive decline into dementia [18].

Another specific subtype of neuronopathic GD includes patients who develop progressive myoclonic epilepsy. Although this presentation of type 3 GD (also referred to as type 3a) was among the first appreciated [35], it is perhaps the least well characterized. Previous case reports of patients with GD and myoclonic epilepsy have been scattered, and often the myoclonus is not an emphasized feature. There appears to be variability in the age of presentation as well as the rate of disease progression in affected individuals [36]. 

### 3.4. Overlapping Phenotypes

While patients with neuronopathic GD are typically divided clinically into two groups, the phenotypes encountered represent a continuum, ranging from the most severe perinatal cases to mild involvement with oculomotor abnormalities [9], and the wide spectrum of phenotypic variation and genotypic heterogeneity is characteristic of all types. Although genotype may play a role in determining the degree of neurological involvement, the extent and mechanisms by which a defined genotype leads to a particular phenotype still remain unknown.

A major challenge to clinicians when GD is identified in the first year of life is to attempt to predict prognosis. This is essential for counseling and for therapeutic decision-making. While at times the genotype may specifically be suggestive of types 2 or 3 GD, in other instances it can be difficult to make predications at the time of diagnosis. One specific research finding may prove helpful in these situations. Based on early studies of a type 2 GD mouse, it was recognized that patients with type 2 GD had distinct epidermal abnormalities [37]. Glucocerebrosidase plays an essential role in maintaining epidermal barrier function by regulating the generation of ceramides from glucosylceramides in the stratum corneum of skin [38]. Evaluating skin ultrastructure in a cohort of 22 children with type 2 GD, it was noted that these babies displayed characteristic electron dense, non-lamellar clefts and immature-lamellar membranes not present in controls and subjects with type 1 GD. Thus, alterations in epidermal ultrastructure may provide a needed tool to distinguish type 2 GD from other subtypes [39].

## 4. Neuropathology

Wong et al. describe specific and unifying neuropathological features across the spectrum of clinical types of GD [40]. Features common to all types include perivascular lipid-laden macrophages (Gaucher cells) and perivascular or region-specific gliosis. The gliosis is seen in CA2–CA4 of the hippocampus, layer 3 and 5 for parietal cortex and layer 4b of the occipital cortex. Neuronal loss is also noted in these regions in patients with neuronopathic GD [40]. 

## 5. Differential Diagnosis 

Diagnosing and treating patients with GD has always been a challenge to clinicians. The average delay from clinical onset to diagnosis can range from 3.5 to 7 years. The differential diagnosis of GD includes other lipid accumulation abnormalities such as Niemann–Pick disease, Tay–Sachs disease, and Pompe disease. Patients presenting in the pediatric period are often first felt to have a hematologic malignancy because of the cytopenia and organomegaly. Moreover, Gaucher-like cells can be found in hematologic disorders such as chronic myelogenous leukemia, acute myeloid leukemia, chronic lymphocytic leukemia, Hodgkin lymphoma, multiple myeloma, and idiopathic thrombocytopenia (Table 1).

### Treatment Strategies for Neuronopathic Gaucher Disease (Table 2)

While some patients with neuronopathic GD can benefit from ERT because of its effect on hematologic and visceral symptoms, it has no impact on brain involvement because it does not cross the blood–brain barrier. An attempt was made to directly administer enzyme into the brain using convection-enhanced delivery in several animal models and human subjects. While the therapeutic enzyme was successfully delivered into the brains of small and large animals, and then into the brainstem of a patient with Gaucher type 2, no clinical improvement ensued [41]. Currently, researchers are exploring whether tagging of therapeutic proteins with blood–brain barrier-crossing peptides or loading them into exosomes could enhance delivery to the brain. It remains to be tested if these strategies could indeed render ERT suitable for treatment of neuropathic forms of GD [42,43]. Furthermore, while current SRT drugs on the market do not show efficacy in the brain, a novel SRT compound named Genz-682452 appeared to reduce storage and alleviate neurological symptoms in neuropathic GD mouse models [44]. This compound is likely to be explored further in patients with neuronopathic GD. Chemical chaperones are a third strategy for the treatment of GD that might potentially impact neuronopathic GD. Such small molecules can bind the mutant protein in order to facilitate its transfer to lysosomes [45]. Both inhibitory and non-inhibitory compounds have been identified that enhance enzymatic activity. Using induced pluripotent stem cell-derived models of GD, Aflaki et al demonstrated that two of these small molecule non-inhibitory chaperones could successfully restore enzyme activity, even in type 2 macrophages and neurons, and reverse glycolipid storage [46,47]. One of the compounds was shown to have a wide bio-distribution, including penetrance in the brain when injected in mice. Further work is in progress to optimize these lead compounds. 

Another potential chaperone for glucocerebrosidase is ambroxol, a drug marketed as an expectorant. It was found through a drug screen to be a mixed inhibitor of glucocerebrosidase, and its potency has been shown in cell, murine and drosophila models [48,49,50]. One preliminary study conducted in Japan was performed on 12 patients with type 1 GD and five with type 3 GD. The results indicated no further deterioration of symptoms in the patients with type 3 GD and the authors reported some improvement of myoclonus and pupillary light reflex in patients [51]. Clearly, further evaluations are warranted. 

Attempts have been made to use gene therapy for the treatment of GD. Dunbar et al utilized a gene transfer protocol on GD3 patients, with the aim of introducing the *GBA1* gene into hematopoietic cells and then injecting the corrected cells into affected patients. Results were disappointing as the GCase levels proved too low for any clinical effect [52]. Since then, lentiviral vector gene transfer techniques have been used in mouse models of GD with promising results, however this approach is still at its preliminary stages [53].

Bone marrow transplantation (BMT) has been undertaken in individuals with severe GD, primarily those with chronic neurological involvement. Successful engraftment can correct the metabolic defect, improve blood counts, and reduce increased liver volume [54]. In a few individuals, stabilization of neurological and bone disease has occurred. However, the morbidity and mortality associated with BMT limit its use in individuals with type 1 and type 3 GD. Therefore, this procedure has been largely superseded by ERT.

Designing treatments for type 2 GD has other inherent difficulties. It is not clear if anything will be able to reverse the brain damage once it has manifested. Pre-symptomatic diagnosis or in utero therapy may be necessary to mitigate the neuronal loss. A better understanding of the pathogenesis of this form of the disease may facilitate the development of improved therapeutics. Clinically, these patients need coordinated evaluations by a multidisciplinary team that can assess the patient’s rapidly changing care needs. 

## 6. Ongoing Challenges

Despite the elucidation of the *GBA1* gene defect and relevant biochemical pathways in GD, the mechanisms by which substrate accumulation in various cells causes disease manifestations is still not completely understood [55]. Multiple studies are currently underway to understand the pathophysiology, particularly neuronal involvement. A better understanding of these features may lead to the development of more effective treatments for neuronopathic forms of this disease. The recent discovery of the Gaucher–Parkinson’s association has now re-focused research towards delineating the role of glucocerebrosidase in the brain. Such insights may eventually benefit patients with neuronopathic GD, as well as those with more common idiopathic Parkinson’s disease. The observed reciprocal relationship between glucocerebrosidase activity and α-synuclein levels may aid in the development of new therapeutics for the treatment of both GD and PD.

## 7. Conclusions

An accurate and timely diagnosis of GD is critical for genetic counseling, patient management and family planning, and can inform future pregnancies. However, the phenotypic heterogeneity observed in these patients makes the diagnosis a challenge for physicians who are unfamiliar with the disease. Moreover, patients with neuronopathic GD have several difficult counseling issues, which could potentially impact their management. The broadening spectrum, as well as the overlap of phenotypes associated with neuronopathic GD, complicates discussions concerning prognosis and the potential benefits of therapy. By unraveling the factors contributing to heterogeneity in this disorder, we may be able to have a direct impact on studies of pathophysiology and therapeutic options available.

## Figures and Tables

**Table 1:** Disorders to be Considered in the Differential Diagnosis of Gaucher Disease (GD).

No.	Disorders
1.	Niemann Pick disease
2.	Tay–Sachs disease
3.	Pompe disease
4.	Chronic Myelogenous Leukemia
5.	Acute Myeloid Leukemia
6.	Chronic Lymphocytic Leukemia
7.	Hodgkin Lymphoma
8.	Multiple Myeloma
9.	Idiopathic Thrombocytopenia

**Table 2:** Management strategies for neuronopathic Gaucher. ERT: enzyme replacement therapy; SRT: substrate replacement therapy.

Strategy	Route	Comments
ERT Imiglucerase Taliglucerase alpha Velaglucerase alpha	Intravenous	First line of treatment To provide sufficient amount of glucocerebrosidase, allowing the processing of glucosylceramide Reduction of spleen and liver size Resolution of anemia and thrombocytopenia Prevents acute bone crises and fractures Improvement of bone mineral density Does not cross the blood–brain barrier and has no impact on neurological disease
SRT Eliglustat Miglustat	Oral	Second line of treatment, if ERT is not tolerated To minimize the accumulation of glucosylceramide within cells by inhibiting glucosylceramide synthase Reduction of spleen and liver size Resolution of anemia and thrombocytopenia Prevents acute bone crises and fractures Improvement of bone mineral density Does not cross the blood–brain barrier and has no impact on neurological disease
Chaperones Isofagamine Ambroxol Bicyclic L-idonojirimycin Other non-inhibitory chaperones	Oral	Development of this type of treatment is still in the early stages Clinical trials have yet to be conducted Can cross the blood–brain barrier, which opens up the possibility of treating neurological symptoms that are not responsive to ERT
Bone Marrow Transplant	-	Corrects the metabolic defect Improve blood count Reduces increased liver volume. In a few individuals, possible stabilization of neurological and bone disease was reported Significant morbidity and mortality and therefore is not currently recommended for the management for neuronopathic GD Replaced by the use of ERT
Gene Therapy	-	Potential therapeutic approach Lentiviral vector gene transfer techniques have been used in mouse models with promising results Still in preliminary stages
